# Targeting Glioma Stem Cells: Therapeutic Opportunities and Challenges

**DOI:** 10.3390/cells14090675

**Published:** 2025-05-06

**Authors:** Asma Mahdi, Mohamed Aittaleb, Fadel Tissir

**Affiliations:** College of Health and Life Sciences, Hamad Bin Khalifa University (HBKU), Education City, Doha P.O. Box 5825, Qatar; asma49687@hbku.edu.qa (A.M.); maittaleb@hbku.edu.qa (M.A.)

**Keywords:** glioma stem cells, therapeutic targets, plasticity, quiescence, therapy resistance

## Abstract

Glioblastoma (GBM), or grade 4 glioma, is the most common and aggressive primary brain tumor in adults with a median survival of 15 months. Increasing evidence suggests that GBM’s aggressiveness, invasiveness, and therapy resistance are driven by glioma stem cells (GSCs), a subpopulation of tumor cells that share molecular and functional characteristics with neural stem cells (NSCs). GSCs are heterogeneous and highly plastic. They evade conventional treatments by shifting their state and entering in quiescence, where they become metabolically inactive and resistant to radiotherapy and chemotherapy. GSCs can exit quiescence and be reactivated to divide into highly proliferative tumor cells which contributes to recurrence. Understanding the molecular mechanisms regulating the biology of GSCs, their plasticity, and the switch between quiescence and mitotic activity is essential to shape new therapeutic strategies. This review examines the latest evidence on GSC biology, their role in glioblastoma progression and recurrence, emerging therapeutic approaches aimed at disrupting their proliferation and survival, and the mechanisms underlying their resistance to therapy.

## 1. Introduction

Glioblastoma (GBM), or grade 4 glioma, is a common, aggressive, and lethal primary brain tumor in adults, accounting for around 50% of malignant brain tumors. Patients diagnosed with GBM have a poor prognosis and a median survival of 15 months [1]. Despite the efforts to alter the course of the disease, tumor recurrence inevitably overcomes the conventional therapy consisting of surgery, radiotherapy (RT), and chemotherapy. Management of recurrent glioblastoma remains unsuccessful [2,3], underscoring the urgent need for improved therapeutic strategies in addressing this devastating disease. A myriad of factors could potentially lead to recurrence, which makes the fight against the disease very challenging and impedes the ongoing efforts to improve the patients’ outcomes. One critical step in the conventional treatment is the surgical resection of the tumor. However, the pre-operation imaging techniques, including the most advanced MRI and PET technologies, fail to fully detect the infiltrative edges of GBM, making it difficult to completely remove the tumor during resection. Another challenge is the extreme vulnerability of the brain tissue [4]. Recurrence partly arises from resistant residual tumor cells left from incomplete resection or from differentiation of quiescent glioma stem cells (GSCs). In both cases, recurrence requires cancer cells to proliferate and invade surrounding brain tissue, while resisting therapy [5,6,7]. Thus, new therapeutic approaches against residual GSCs are needed to prevent GBM survival and recurrence. Impairing proliferation and growth of GSCs constitutes an opportunity to restrain GBM and prevent relapse. In this manuscript, we will give an overview of current attempts to contain GBM, with a particular focus on GSCs, and discuss the challenges that still hinder its treatment.

## 2. Origin and Role of GSCs

Significant progress has been made in understanding advanced-stage GBM, leading to its classification into well-defined molecular subtypes at both bulk and single-cell levels [8,9,10]. However, the early stages of glioma genesis and the underlying etiology of the tumor remain elusive due to the challenges associated with early detection and limited access to early-stage tumor samples. Accumulating evidence suggests that solid tumors originate from cancer stem cells (CSCs), a fraction of cancer cells with high self-renewal, proliferation, and differentiation capabilities [11]. This concept was first introduced in acute myeloid leukemia (AML), where studies demonstrated that a subset of leukemia stem cells drives tumor initiation and progression [12]. Subsequently, CSCs have been identified in various other cancers, including breast [13], prostate [14], colorectal [15], and pancreatic cancers [16]. In GBM, GCSs isolated from tumor samples exhibit tumorigenic potential as they can form neutrospheres, indicating their self-renewal abilities [17,18].

**Figure 1:**
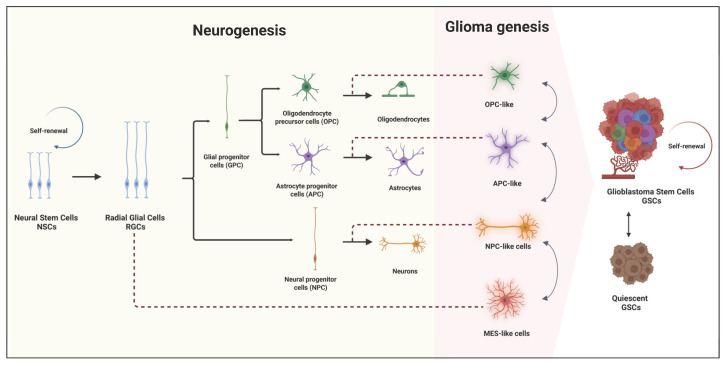
Similarities between neurogenesis and glioma genesis: implications for GBM cell states and cells of origin. During neurogenesis, neural stem cells (NSCs) differentiate into radical glia cells (RGCs), which give rise to two types of committed progenitor cells, namely the neural progenitor cells (NPCs) and glial progenitor cells (GPCs). Subsequently, NPCs produce neurons, whereas GPCs diversify into oligodendrocytes progenitor cells (OPCs) that generate oligodendrocytes and astrocyte progenitor cells (APC) that generate astrocytes [40]. Single-cell RNA analysis of GBM revealed the presence of NPC-like, OPC-like, and APC-like cell states, which have similar transcriptional features as NPCs, OPCs, and APCs, respectively. The mesenchymal-like (MES-like) cell state has relatively more distant similarities with RGCs [24].

GBM was believed to originate from the dedifferentiation of mature neural cells into the progenitor state that initiates and maintains the tumor progression [19]. However, this hypothesis was questioned with the discovery of adult neural stem cells [20] and the identification of intriguing similarities (molecular signatures and signaling pathways) between NSCs and GSCs [17,18,21] (Figure 1). These findings suggest that the cells of origin for glioblastoma may be NSCs that undergo malignant transformation, transitioning into GSCs capable of driving neoplastic progression in the brain. In line with that, PDGF activation in NSCs in the subventricular zone (SVZ) is sufficient to induce hyperplasia with early stages of tumor formation hallmarks [22]. Furthermore, the molecular classification of high-grade gliomas (HGG) has provided critical insights into their progression and resemblance to distinct stages of neurogenesis, emphasizing the intricate relationship between glioma genesis and normal neurogenesis [23]. These cells were isolated using the neural stem cell marker CD133 and, when cultured, differentiated into tumor cells that closely resemble the original patient tumor [17].

Modern and advanced technologies such as single-cell RNA sequencing (scRNA-seq) and RNA velocity analysis were instrumental in grasping the dynamics of cellular transitions underlying tumor initiation and mapping its developmental trajectories. These analyses confirmed that tumor evolution follows conserved neurodevelopmental programs and that rapidly cycling cancer cells are the most tumorigenic and resistant to therapy [24,25,26]. Tissue samples from GBM patients and genome-edited mouse models uncovered low-level GBM driver mutations in normal SVZ tissue distant from the tumor mass [27]. These mutations, present at high levels in corresponding tumors, suggest that NSCs carrying driver mutations migrate from the SVZ to other brain regions, contributing to malignant glioma development [27]. Malignant glioma can arise from p53 loss in combination with other mitogenic signaling pathways. Notably, mutant p53 proteins in NSCs promote the accumulation of additional oncogenic alterations, triggering the expansion of Olig2(+) progenitor-like cells and initiating glioma formation [28]. Moreover, several genes involved in cell-cycle regulation and mitotic progression of neural stem and progenitor cells during development are associated with proliferative progenitor cells in GBM. Examples include Aurora kinase A [29], Forkhead Box M1 (FOXM1) [30], and Diaphanous-related formin 3 (DIAPH3) [31,32,33]. This disruption of mitotic regulation in neural progenitor cells leads to chromosomal instability, which could induce neoplastic transformation. Glioma genesis and invasiveness may be driven by the accumulation of genetic mutations and dysregulation of signaling pathways involved in the proliferation and differentiation of NSCs [34]. Multiple lines of evidence support this hypothesis: (1) the combined activation of Ras and Protein Kinase B (AKT) pathways in neural progenitors in mice, but not in differentiated astrocytes, induces HGG with the histological characteristics of human GBM [35]; (2) a two-gene prognostic model suggests AKT and NOTCH signaling are critical for distinguishing outcomes in gliomas [36]; (3) GSCs exhibit elevated WNT activity, enhanced sphere-forming potential, and increased SOX2 expression [37]; (4) aberrant Wnt/β-catenin signaling promotes glioma aggressiveness and drug resistance by inducing epithelial-mesenchymal transition (EMT) through upregulation of Fos-related antigen-1 (FOSL1) [38]; and (5) spatial transcriptomic analysis showed that NOTCH signaling is upregulated in mesenchymal-like GBM cells residing in infiltrated brain tissue [39].

## 3. Therapeutic Opportunities Targeting GSCs

The treatment of GBM is challenging, and its cure remains unattainable. The mainstay therapy has not changed since 2005. It consists of surgical resection (introduced by Rickman Godlee in 1884), supplemented by adjuvant treatments that include RT [41] and alkylating agent temozolomide (TMZ) [2,42]. Adding adjuvants to clinical care prolongs the patients’ survival by a few months but decreases the quality of life because of the systemic toxicity of RT and chemotherapy. In addition, many tumors either exhibit or acquire resistance during the treatment [43]. The use of anti-angiogenic antibodies (e.g., Bevacizumab, aka Avastin), which target vascular endothelial growth factor (VEGF), led to an increase in progression-free survival (PFS) [44] but did not improve the overall survival (OS) of patients [45,46]. Hence, many challenges persist, such as the difficulty of removing all tumor cells because of their intrinsic nature to infiltrate normal brain tissue and cause tumor relapses and the blood–brain barrier (BBB), which limits drug delivery. Innovative and promising approaches are being investigated to overcome these challenges. They are aimed at (1) a better understanding of the pathophysiological mechanisms driving glioma growth and recurrence, (2) the discovery of new means and tools to selectively target tumor cells while preserving healthy ones, (3) the translation of the new knowledge into novel practices in neurosurgery and neuro-oncology, (4) the development of additional/better preclinical models, and (5) the use of personalized treatments (precision medicine) in clinical trials. In the next section, we will review the current and emerging treatments focusing on strategies that control the proliferation and survival of GSCs.

### 3.1. Targeting GSCs Through Genetic Mechanisms

Cell signaling pathways that promote cell proliferation, growth, or survival have oncogenic potential. On the other hand, pathways that restrict these processes have an oncosuppressor potential. These pathways offer therapeutic options for GSC treatment. Receptor tyrosine kinases (RTKs) are transmembrane receptors implicated in multiple biological processes, including proliferation, differentiation, motility, and metabolism. RTKs are activated by different growth factors that bind to their extracellular region, inducing receptor dimerization/oligomerization and intracellular tyrosine kinase activity [47]. RTKs’ ligands comprise Epidermal Growth Factor (EGF), Fibroblast Growth Factor (FGF), Vascular Epidermal Growth Factor (VEGF), Platelet-Derived Growth Factor (PDGF), Transforming Growth Factor (TGF), and Hepatocyte Growth Factor (HGF). Activated/autophosphorylated RTKs regulate a large assortment of key signaling pathways, such as PI3K/AKT/mTOR, RAS/MAPK, and JAK/STAT, thereby conveying cell proliferation and migration signals from the extracellular to the intracellular milieu. The level of RTK activity is tightly regulated by diverse molecules and mechanisms, including autoinhibition and activity of tyrosine phosphatases [48,49]. RTKs acquire oncogenic ability via disruption of the balance between cell proliferation and death in cancer cells [50]. Several RTKs have abnormal expression/activity, mutations, or copy number alterations in GBM. Around a hundred RTK inhibitors have been developed, and several dozens were used in clinical trials in the context of GBM treatment (Table 1). Several multi-targeting RTK inhibitors have been tested either alone or in combination with other therapies, yielding mixed yet inconclusive results. Among these, Anlotinib showed promising outcomes in terms of efficacy and tolerance in a clinical trial of newly diagnosed cases of GBM with unmethylated O6-Methylguanine-DNA Methyltransferase (MGMT) promoter [51], Cabozantinib showed some activity in patients with recurrent GBM who were not treated with antiangiogenic therapies, but did not meet the statistical significance for success [52]. Dasatinib, either alone or in combination with cyclohexyl-chloroethylnitrosourea (CCNU), did not improve the outcome in patients with recurrent glioblastoma [53]. The combination of Dasatinib and Bevacizumab did not improve the outcome as compared to Bevacizumab alone [54]. Nintedanib was active against recurrent GBM [55]. Pazopanib showed biological activity in situ but did not prolong PFS [56]. Pexidartinib was not effective either as single therapy in recurrent glioblastoma patients [57], or in combination with RT and TMZ in newly diagnosed GBM patients [58]. Ponatinib showed minimal activity in bevacizumab-refractory GBM patients [59]. Regorafenib showed moderately encouraging outcomes as a second-line treatment in recurrent GBM patients [60], Sunitinib exhibited little to no activity in GBM [61]. Vandetanib was well tolerated but did not significantly change the overall survival (OS) [62]. In the next section, we will elaborate on strategies used to control the proliferation and survival of GSCs via Epidermal Growth Factor Receptor (EGFR), Fibroblast Growth Factor Receptor (FGFR) and Hepatocyte Growth Factor Receptor (HGFR) modulation.

EGFRs are the most frequently affected growth factor receptors in GBM. Around 60% of cases have driver mutations, rearrangements, alternative splicing, or amplifications in EGFR [126,127,128]. Therefore, the inhibition of EGFR has been a chief objective in GBM therapy. Targeting EGFR using monoclonal antibodies (e.g., cetuximab, nimotuzumab) or small molecule inhibitors (e.g., Gefitinib, Erlotinib, Dacomitinib, Osimertinib, Depatuxizumab, Mafodotin) has been extensively investigated. Depatuxizumab mafodotin demonstrated no OS benefit in treating newly diagnosed GBM patients with EGFR amplification [68]. Gefitinib was the first FDA-approved EGFR inhibitor. However, its use did not benefit in clinical trials neither alone nor in combination with other treatments [70,71,73,74,129]. Erlotinib, another EGFR inhibitor, was tested in combination with RT and TMZ with conflicting results in primary glioblastoma [130,131,132,133], and some beneficial outcomes in recurrent glioblastoma [134,135,136,137,138,139,140,141,142]. Dacomitinib is a second-generation EGFR inhibitor that showed promising results in pre-clinical models [75] but did not provide any substantial clinical benefit when tested in patients [76,77]. Osimertinib is a third-generation EGFR inhibitor with higher potency for inhibiting EGFR activity [79]. A case report on a young female patient (with EGFR amplification and multifocal progression of the disease after surgery, RT, TMZ, and bevacizumab) who was treated with Osimertinib daily for one month, showed a complete response in one of the tumor sites [80]. A retrospective study showed that 46.7% of patients treated with osimertinib and bevacizumab have PFS after 6 months [81]. Overall, EGFR inhibitors exhibit reduced efficacy in GBM therapy. Novel approaches in the field focus on targeting EGFR in combination with the androgen receptor (AR), which may be activated in a ligand/hormone-independent manner via EGFR signaling in GBM cells [143]. AR expression is positively correlated with EGFR expression in patients [143]. Enzalutamide, an AR inhibitor, decreases the density of cancer stem cell populations and improves survival in a glioblastoma orthotopic mouse model [144]. Combined with Afatinib (ErbB family inhibitor), Enzalutamide has a greater impact on GBM cell death.

FGFRs are involved in 3% of GBM cases only [128]. Nevertheless, chromosomal translocation-mediated fusion between FGFR1/FGFR3 and transforming acidic coiled-coil (TACC) protein generates FGFR-TACC proteins with constitutive active kinase activity in GBM cells [145]. FGFR3 is the family member that is mostly affected by amplification and fusion in GBM [146,147]. Analysis of GBM data from The Cancer Genome Atlas (TCGA) GBM revealed that FGFR2 is frequently deleted, and several FGFR2 fusion proteins (e.g., CXCL17–FGFR2 and SIPA1L3–FGFR2) have been identified. These chimeric proteins do not seem to be functional due to a disrupted kinase activity [148]. Nonetheless, a case of FGFR2 amplification with an FGFR2-TACC2 fusion protein in a patient with an aggressive form of GBM has been reported [149]. Compared to EGFR and VEGFR inhibitors, the identification of FGFR inhibitors was slow to emerge. Some available RTK inhibitors were reformulated to increase FGFR inhibitory activity. These include Erlotinib, Sorafenib, Lapatinib, Ponatinib, Lucitanib, and Nintedanib. A few FGFR-specific inhibitors are currently being developed and/or validated. Among the FGFR selective inhibitors, Erdafitinib showed a robust benefit and decelerated tumor growth in GBM with FGFR3-TACC3 fusion [69]. In a 53-year-old man with a recurrent GBM and FGFR3-TACC3 fusion, Pemigatinib induced a partial response and neurological improvement [82]. Finally, Infigratinib (NVP-BGJ398) showed limited efficacy in monotherapy for patients with recurrent gliomas and different FGFR alterations [150].

HGFR/c-Met is a single receptor tyrosine kinase activated by HGF/Scatter Factor (SF). HGFR plays essential roles in the biology of GSCs [151,152], even though HGFR amplification occurs in less than 5% of GBM cases [153]. Capmatinib is a selective HGFR inhibitor that entered clinical trials but had little activity in PTEN-deficient recurrent GBM [136]. Trials combining capmatinib and bevacizumab are currently in progress (https://www.mycancergenome.org/content/clinical_trials/NCT02386826/ accessed on 1 May 2025). Crizotinib, another selective HGFR inhibitor, had promising results in phase I clinical trials in patients diagnosed with GBM. These patients treated with Crizotinib in addition to RT and TMZ presented higher OS and PFS than patients who received RT and TMZ only [96]. Crizotinib and erlotinib (an EGFR inhibitor) showed synergistic effects in orthotopic tumor model mice [97], and it is tempting to propose the combination of Crizotinib and erlotinib in patients with HGFR+/EGFR+ GBM.

Another major intracellular signaling pathway that regulates GSC proliferation is the PI3K/mTOR, which integrates several environmental stimuli such as nutrients, hormones, and growth factor stimulation. PI3K activation mediates the conversion of phosphatidylinositol-4,5-bisphosphate into phosphatidylinositol-3,4,5-triphosphate (PIP3). PIP3 recruits additional kinases such as 3-Phosphoinositide-Dependent Protein Kinase-1 (PDK1) and AKT. AKT activation regulates the cell cycle and apoptosis by phosphorylating the (1) Forkhead Box O (FOXO) transcription factors, thus reducing expression of proapoptotic and cell cycle-regulatory genes [154], (2) pro-apoptotic proteins Bcl-2 Antagonist of Cell Death (BAD), Bcl-2-Associated X Protein (BAX), and caspase-3/9 [155], (3) MDM2, an oncoprotein that translocates to the nucleus where it binds p53 targeting it for degradation [156], (4) cell-cycle proteins p27 and p21, stabilizing cyclin D1/D3 and promoting cell growth [72], (5) glycogen synthase kinase (GSK)-3 β [157], and (6) IκB Kinase Alpha (IKKα) and Tumor Progression Locus 2 (Tpl2), thus activating the oncoprotein Nuclear Factor Kappa B (NF-κβ). Finally, in addition to directly regulating several cellular processes involved in cell transformation, AKT also activates mTORC1, another master regulator of tumorigenesis [158]. The PI3K/ mTOR pathway orchestrates interconnected signaling cascades that converge on GSC proliferation and death. Over 80% of GBM have genetic alterations in RTK/PI3K [159] making it a target choice in GBM therapy. There are dual PI3K/mTOR inhibitors, pan-PI3K inhibitors, and isoform-specific inhibitors.

Dactolisib, Voxtalisib, and paxalisib are dual PI3K/mTOR inhibitors that have been investigated. In GBM cells and in an orthotopic rat model, Dactolisib combined with radio and chemotherapy promoted anti-tumor activity in vitro and inhibited tumor growth and prolonged survival in vivo [98]. In mice, however, Dactolisib alone did not inhibit tumor growth or offer any benefit for survival despite considerable side effects [99]. Dactolisib is currently being trialed in glioblastoma patients (NCT02430363, https://clinicaltrials.gov/study/NCT02430363?tab=history&a=1 accessed on 1 May 2025). Voxtalisib has been tested in a phase I clinical study. It showed acceptable levels of safety but limited anti-tumor activity in GBM patients [100]. Paxalisib (GDC-0084) reduced tumor growth in orthotopic mouse models [101], and is showing promising results in clinical trials for newly diagnosed and recurrent glioblastoma alike [102,103,104].

Buparlisib, Pilaralisib, Pictisilib, and Sonolisib are pan-PI3K inhibitors. Buparlisib entered clinical trials of newly diagnosed GBM but did not pass phase 1 because of excessive toxicity [105]. In recurrent GBM, Buparlisib showed minimal to no efficacy in phase II clinical trials [160]. Bevacizumab-naïve recurrent GBM patients treated with Buparlisib in combination with Bevacizumab had similar clinical outcomes to the patients treated with bevacizumab alone [106]. Pilaralisib [100] and Pictisilib [107] are in phase II clinical trials for recurrent glioblastoma. Sonolisib (PX-866) was well tolerated in a phase 2 clinical trial. However, the overall response rate was low, and the study failed to meet primary endpoints [108]. Given that p110α, β, and δ isoforms are differentially expressed in GBM, inhibitors targeting these isoforms may prove astute. Various inhibitors have been developed, or are under development, but have not been extensively utilized in clinical trials. GSK2636771, a p110β-specific inhibitor, is currently employed in clinical settings on patients with PTEN-deficient advanced solid tumors (NCT01458067, https://clinicaltrials.gov/study/NCT01458067 accessed on 1 May 2025). However, the results have not yet been published.

Rapamycin was the first mTOR inhibitor to be used in clinical research. Analogs of rapamycin, such as Everolimus, ridaforolimus, Sirolimus, and Temsirolimus, were developed and used in GBM clinical trials. Everolimus with RT/TMZ was well tolerated in phase 1 but did not improve the survival of patients in phase 2 clinical trials [109]. Likewise, Tesirolimus did not offer any significant benefit to patients even though two studies suggested that patients with phosphorylated mTOR [110] or phosphorylated AKT [111] may benefit from the treatment. Clinical trials using Sirolimus (NCT00047073) and Ridaforolimus (NCT00087451) were not more successful, likely because these inhibitors do not target mTORC2 or block the PI3K/AKT/mTOR signaling. A second generation of ATP-competitive inhibitors, targeting mTORC1 and 2 (e.g., Vistusertib, Torin1, and Torin2), have been developed but have not been evaluated in clinical trials yet.

### 3.2. Targeting GSCs Through Epigenetic Mechanisms

Epigenetic changes comprise DNA methylation, histone modifications, and chromatin remodeling. These changes alter gene expression, thus reducing tumor suppressor pathways, promoting oncogenic pathways, and/or conferring plasticity to cancer cells. Methylation of genomic regulatory regions (mediated by DNA methyltransferase DNMT) can silence tumor suppressor genes or genes involved in DNA damage repair and, therefore, cell death, whereas global DNA hypomethylation can activate oncogenes and trigger genome instability [161]. In line with this, large subsets of GBM tumors are characterized by global hypomethylation. In contrast, hypermethylation of CpG islands has been found at the promotor of the BCL2L11 gene encoding the pro-apoptotic protein BIM [162], and high levels of BIM were associated with resistance of GBM cells to EGFR inhibition-mediated treatment [163]. Finally, methylation of the MGMT gene, which encodes the DNA repair enzyme O6-methylguanine DNA methyltransferase, correlates with better response to TMZ and prolonged survival and is, therefore, a favorable prognostic factor in GBM [164]. DNMT inhibitors Decitabine (5-aza-2′-deoxycytidine) and 5-azacytidine were explored in experimental models and clinical trials. Decitabine altered DNA methylation status, affecting differentiation in glioma cells [112]. It also increased neo-antigen expression and susceptibility to T-cell responses of GBM cells in vitro [113]. 5-azacytidine reduced the viability of human GBM cells in vitro and delayed the growth of glioblastoma in vivo in orthotopic models [114].

Another epigenetic mechanism involves posttranslational histone modifications such as methylation and (de)acetylation. Histone methyltransferases (e.g., Enhancer of Zeste Homolog 2 [EZH2]) amend the expression of important genes such as PTEN and NFκβ in GBM [165,166]. EZH2 acts as a subunit of the polycomb repressive complex 2 (PRC2) and mediates methylation of lysine27 of histone H3 (H3K27). Alterations in H3 K27, particularly a lysine-to-methionine mutation, are associated with the pathogenesis of diffuse midline glioma, a lethal pediatric HGG. Thus, EZH2 has been suggested as a potential therapeutic target. Deacetylation of histones triggers a closed conformation of chromatin and reduces the expression of tumor suppressor genes. Analysis of GBM pre-clinical models revealed a role for histone deacetylase (HDAC)6 in the proliferation of GSCs and resistance to TMZ [167]. HDAC6 regulates EGFR levels through trafficking and degradation [168], through cell motility via the deacetylation of α-tubulin [169], and through the EGFR-Ras-Raf-MEK-ERK signaling [170]. Given their ability to regulate epigenetic signatures, HDAC inhibitors such as Balinostat, Panobinostat, and Vorinostat have received much attention in clinical research. Balinostat was tested in combination with RT and chemotherapy in newly diagnosed GBM [115]. Panobinostat was evaluated together with bevacizumab in recurrent GBM [116]. None of these inhibitors showed any added value for patients. Likewise, Vorinostat has been unsuccessfully trialed as a monotherapy [117], or in combination with Bevacizumab [171], with Bortezomib [172], and with RT and TMZ [173]. Valproic acid, a selective inhibitor for class I/IIa HDACs [118], seems to reduce cancer hallmarks in synergy with other therapeutic medications in pre-clinical models [119]. Newly diagnosed GBM patients treated with valproic acid (together with RT and TMZ) have a significantly longer OS (approximately 30 months) [120]. Valproic acid may potentiate the TMZ efficacy by increasing the availability of DNA to this alkylating agent. This is a considerable improvement, and larger clinical trials are in progress to assess if valproic acid could be added to the GBM standard care regimen. Other HDAC isoform-specific inhibitors, such as KA2507 and JBI-802, have been developed and are being evaluated in clinical trials [121] (NCT05268666, https://clinicaltrials.gov/study/NCT05268666 accessed on 1 May 2025).

## 4. Why Is GBM So Difficult to Defeat?

Despite a better understanding of the disease and hundreds of clinical trials, treatment advances for patients with GBM remain insignificant [174,175]. There are numerous reasons for failure. Chief among these are (1) the extreme delicateness of the brain tissue and infiltrative nature of GBM, which make the complete removal of the tumor virtually hopeless; (2) the presence of BBB, which hampers drug delivery to the tumor site; (3) the absence of scientific tools (e.g., biomarkers) and policies to screen for and detect early stages of the tumor, which might be easier to handle and eradicate; and (4) the inherent characteristics of GBM cells, which enable them to adapt to changing microenvironment and elude therapeutic treatment. In the last section of the review, we will discuss the mechanisms underlying the plasticity and resistance to therapy of GBM cells.

### 4.1. Heterogeneity

GBM is one of the most heterogeneous cancers, characterized by a wide range of tumor cell types, genetic mutations, and distinct phenotypic features [176]. This heterogeneity complicates diagnosis and treatment and contributes to disease aggressiveness. Within GBM, GSCs play a significant role in driving heterogeneity through their molecular profiles and cellular behaviors. GSCs exist in different states according to environmental cues, further amplifying the diversity within the tumor [177]. This dynamic and multifaceted heterogeneity of GSCs is a major barrier to developing effective therapies, as targeting one subset of cells may fail to address the diverse population present in the tumor [178].

Mutations in genes such as EGFR, PDGF Receptor Alpha (PDGFRA), and Neurofibromin 1 (NF1) are considered driver mutations that define distinct clonal populations and contribute to tumor heterogeneity [8,179]. According to their transcriptomic profile, GBM tumors were classified into three major subtypes: classical (characterized by amplification/mutation in EGFR), proneural (characterized by alterations in PDGFRA or by homozygous deletion of CDKN2A), or mesenchymal (characterized by deletions in NF1 or PTEN) subtypes. These subtypes are characterized by distinct prognostic outcomes, molecular profiles, and biological behaviors [9,180,181]. From another side, as tumor cells continue to divide, they also accumulate passenger mutations, introducing additional genetic variation and generating sub-clonal progenies [182]. In response to treatment, tumor cells can develop new mutations reflecting divergent genetic evolution under therapeutic pressure [183]. Advancements in single-cell sequencing and lineage tracing have further refined the classification of GBM tumors [184]. These studies have provided new insights into the intratumoral heterogeneity, revealing that at the single-cell level tumor cells exhibit transcriptional signatures that resemble those of neural progenitor cells (NPCs), oligodendrocyte precursor cells (OPCs), astrocytes (ACs), and mesenchymal (MES) cells. As a result, GBM cells are now classified into NPC-like, OPC-like, AC-like, and MES-like malignant cell states (Figure 1). This more nuanced classification reflects the complex and dynamic nature of GBM, highlighting the variability in cellular states within the tumor and enhancing our understanding of its molecular landscape [10].

### 4.2. Plasticity

Another challenge that stands in the way of understanding the GSC population is the diversity of stem cell markers. Proteins that are enriched in these stem-like cell populations include CD133 (prominin 1) [17], NESTIN [185], OLIG2 [186], SOX2 [187], NANOG [188], integrin α6 [189], CD15 (SSEA-1) [190] and ALDH [191]. These markers are found in higher levels in GSCs; however, no single marker or combination of markers has been discovered that uniquely and fully identifies GSCs [182]. It was previously thought that GSCs follow the hierarchical model of cellular differentiation, which leads to the progression from a multipotent stem clone to differentiated progenies [192,193]. However, advances in time-lapse microscopy, RNA velocity analysis, and in vivo lineage tracing have revealed that GSCs are not confined to these defined states. Instead, they exhibit a dynamic and flexible model of differentiation, transitioning between different cellular identities [193]. These different cell states are triggered and/or selected by factors like environmental niches, treatments, transcriptional programs, and epigenetic influences, leading to the presence of distinct states in particular locations and in a time-dependent manner [26,177,194]. The dynamic flexibility allowing GSCs transition from one state to another is a transitory state within neurodevelopmental (NPC, AC-, or OPC-like cells) and injury response (MES-like) gradients [195]. This transient states allowed for an additional classification that explain this dynamic where stem-like (Ki67+), undifferentiated and progenitor-like (OLIG2+), and differentiated-like (encompassing neoplastic cells exhibiting oligodendrocyte-like, astrocyte-like (EGFR+), and mesenchymal-like (CD44+)) cell states can be also distinguished [196]. By integrating molecular barcodes, single-cell profiling, and mathematical modeling, it is possible to trace and identify the plastic variation in GBM differentiation states, determine their transcriptional identity and quantify the rate and frequency of transitions between these states [197]. This plasticity contributes to enhanced therapy resistance and tumorigenic potential for the GSCs and may offer new targeting opportunities [194,197].

#### 4.2.1. Epigenetic Plasticity

Single-cell RNA sequencing has revealed extensive transcriptional diversity in glioma, often independent of genetic mutations. This plasticity is driven in part by epigenetic changes, which over time reduce DNA methylation levels, especially in gene regulatory regions, leading to chromosomal instability and more aggressive gliomas [198]. These epigenetic changes can be driven by responses to environmental stress, such as hypoxia and irradiation [199]. As a result, GBM cells respond by fluctuating between different cellular lineages, making them difficult to target therapeutically. These regulatory switches, orchestrated by epigenetic changes, drive state transitions that mirror neurodevelopmental pathways. Like neural stem cells, proneural GBM cells use bivalent chromatin to remain multipotent. These cells often show high ten-eleven translocation (TET) enzyme expression, leading to DNA demethylation in key regulatory regions, including RTKs (e.g., EGFR), tumor suppressors (e.g., PTEN, DIAPH3), and oncogenes (e.g., AKT, BRAF). This demethylation disrupts the cell cycle, promoting deregulated cell division [200]. In contrast, hypermethylation drives the differentiation of NPC-like cells into distinct AC-like or OPC-like lineages, restricting their epigenetic and transcriptomic plasticity [200]. Epigenetic methylation regulates neurodevelopment by controlling gene expression and ensuring proper neural differentiation, with its loss leading to brain defects [201].

#### 4.2.2. Transcriptomic Plasticity

GBM cells do not only share epigenetic variation with neurodevelopment but also transcriptomic fluctuations, further increasing plasticity. Transcription factors like ASCL1, HES1, OLIG2, SOX2, and NOTCH-ligands (DLL1/3) play crucial roles in GBM plasticity by maintaining a pool of quiescent glioblastoma stem cells (GSCs) [202,203,204]. While transient transcription factor (TF) expression preserves GSCs, modulates their activity, and promotes a proliferative stem-cell-like state, sustained expression drives differentiation along neurodevelopmental lineages [203]. Prolonged TF expression could induce differentiation and reduce the tumorigenic potential of GBM, offering a promising treatment strategy. Another dynamic phenotype, derived from neurodevelopmental mechanisms, is the migratory pattern of bipolar glioma cells compared to multipolar glioma cells. Just like migrating oligodendrocyte precursor cells (OPCs) and neuroblasts, bipolar glioma cells with fewer microtubes exhibit a more migratory and invasive phenotype. After migration, these cells can transition back to a stationary, multipolar state, allowing them to re-establish cellular networks, demonstrating their adaptive plasticity in the tumor microenvironment [205,206]. These insights suggest that targeting cancer cell plasticity and their dynamic epigenetic and transcriptional states, rather than CSC markers alone, could provide a more effective therapeutic approach.

### 4.3. Quiescence

Necrosis is a hallmark of GBM aggressiveness and is strongly associated with poor prognosis. Rapid tumor expansion outpaces vascular supply, leading to oxygen and nutrient deprivation within the tumor core. Interestingly, while the majority of cells in this region succumb to hypoxia and starvation, a subset of cells located at the periphery of the necrotic zone survives by entering a dormant or quiescent state [207]. Quiescence is a reversible state of cell-cycle arrest that plays an essential role in both normal development and cancer progression [208]. In GBM, quiescent GSCs can enter a dormant G0 phase, enabling them to evade radiation and chemotherapy, which primarily target rapidly dividing cells. This quiescent state contributes to therapeutic resistance, allowing GSCs to survive treatment and re-enter the cell cycle afterward. Upon reactivation, they generate transient populations of highly proliferative cells, driving tumor recurrence [208,209]. Quiescent GSCs are typically characterized by stemness markers like Nestin, Prominin-1 (CD133), and SOX2 and are distinguished by a non-cycling transcriptome profile along with elevated expression of genes involved in cell adhesion, filopodia formation, and cell spreading [210]. These dormant cells, induced by central necrosis at the core of the tumor, are located at the infiltrating edges, where they adopt a mesenchymal-like, migratory morphology, which might explain their capacity to repopulate the brain after treatment [210]. A key characteristic of quiescent cells is their dynamic behavior and reversible state of cell-cycle arrest, allowing them to adapt to the changing microenvironment. Oscillations in protein expression, particularly transcription factors, are critical regulators of the transition between these active and dormant states [211]. It is also important to distinguish between cells that are initially highly proliferative and those that re-enter the cell cycle from quiescent GSCs for a more specific therapeutical targeting after tumor relapses. A synergistic interaction between FOXG1 and Wnt/β-catenin signaling promotes efficient cell cycle re-entry from quiescence [212]. Targeting this mechanism could provide a therapeutic strategy to specifically eliminate the aggressive subpopulation responsible for recurrence. Moreover, BMP signaling drives GSCs into a quiescent state and preserves their self-renewal and tumorigenic potential through p21 expression, simultaneously conferring resistance to RT and chemotherapy [213]. Epigenetic modifications play a role in maintaining the quiescent state of GSCs [214]. These modifications can be targeted to induce differentiation or reactivation of quiescent cells. On the therapeutic side, several attempts have been made to activate “awake” quiescent cancer cells (QCCs) to rapidly enter the cell cycle and hence render them sensitive to anti-proliferative drugs. Along this line, therapy resistant and dormant hematopoietic stem cells were successfully activated upon treatment with IFNα and then eliminated using 5-fluorouracil (5-FU) [215]. IFNα activates these cells in vivo. Another approach is to prevent proliferation of QCCs and maintain them permanently in a dormant state. Modulation of p38 MAPK or ERK1/2 activities [216] and inhibition of WNT and HH pathways [217] have been used to permanently arrest the growth of QCCs and thus prevent tumor relapse. Moreover, additional efforts have been made to selectively eliminate QCCs, taking advantage of their unique characteristics that distinguish them from proliferating cells. Inhibition of IGF-1R reduces the survival of QCCs and cancer recurrence [218]. Despite this progress in QCCs targeted therapy, much remains to be learned about the mechanism of activation and dormancy of QCCs. These efforts are mainly hampered by the lack of a reliable model that recapitulates cancer quiescence in humans and the heterogeneity of these cells.

### 4.4. Resistance to Therapy

#### 4.4.1. Resistance to Chemotherapy

The first-line chemotherapy for GBM is TMZ, which has significantly improved patient survival. However, resistance to TMZ remains prevalent, often resulting in tumor recurrence and treatment failure [219]. TMZ exerts its cytotoxic effects by methylating the O6 position of guanine during DNA replication, leading to base mismatches, G2/M phase arrest, and, ultimately, apoptosis. However, elevated MGMT (O6-methylguanine-DNA methyltransferase) activity repairs alkylated guanine, reducing the efficacy of TMZ and contributing to treatment resistance [164,220]. MGMT expression is significantly elevated in GSCs, conferring strong intrinsic resistance and making these cells highly refractory to therapy [221,222,223]. GSCs expressing MGMT are localized in the tumor’s inner core, where the hypoxia induces fluctuating MGMT expression, contributing to an enhanced protective mechanism against TMZ [224]. Some studies suggest that GSC chemoresistance is linked to an elongated cell cycle through a G2/M arrest regulated by CHK1, CDC25C, and CDC2, allowing more time for DNA repair against TMZ-induced damage [225]. Another mechanism involves the cells entering a quiescent state, allowing them to withstand stress and toxic challenges. Once the cellular damage is repaired, these cells, activated by specific growth factors like cyclin-dependent kinase-2 (CDK2) and E2F, can re-enter the cell cycle [226]. Furthermore, higher expression levels of anti-apoptotic genes, for instance MCL1, BCL-2, and BCL2L1a, have been observed in TMZ-resistant GSC clones compared to differentiated cell lines [222,227].

GSCs often exhibit multidrug resistance (MDR), making them highly resistant to standard chemotherapy. MDR is primarily driven by the overexpression of drug efflux transporters, such as ATP-binding cassette (ABC) transporters, which drain chemotherapeutic agents out of the cell, reducing the accumulation and efficacy of drugs [228,229]. ABCG2 (also known as BCRP1) is highly expressed in GSCs and strongly linked to the side population phenotype, a group of stem-like cells with high drug efflux capacity that are associated with tumor recurrence due to their ability to survive treatment [222,230,231]. MDR1, another ABC transporter, is highly expressed in GSCs, contributing to enhanced drug resistance to anti-cancer drugs such as doxorubicin and etoposide [228,232]. Combining chemotherapeutic drugs with inhibitors of MDR proteins, like melatonin [122] and Perifosine [123], increases the drug sensitivity and enhances the efficacy of the treatment [122,123]. GSCs also utilize signaling pathways, such as the NOTCH and Sonic Hedgehog (SHH) pathways, which are activated by TMZ treatment, resulting in increased expression of genes like NOTCH1 and GLI1, contributing to the chemo-resistant characteristics of GSCs. Inhibiting both pathways using cyclopamine and γ-Secretase inhibitor 1 (GSI-1) significantly boosts TMZ-induced cytotoxicity, presenting a promising strategy to enhance TMZ sensitivity and treatment efficacy [124].

#### 4.4.2. Resistance to Radiotherapy

RT is one of the most used strategies in treating cancer, yet radioresistance continues to be a major obstacle, especially in glioblastoma [233]. The response of tumor cells to radiation and their ability to escape cell death is influenced by several factors, including cell type, radiation dose, and the tumor microenvironment [234]. GSCs are major contributors to radioresistance, allowing them to survive treatment, adopt a more proliferative phenotype, and drive tumor recurrence [235,236]. These cells exhibit a preferential activation of the DNA damage checkpoint response and enhanced DNA damage repair (DDR) capacity through activation of key checkpoint kinases, such as CHK1 and CHK2. The delayed cell-cycle response in GSCs allows them to avoid radiation-induced death and survive [6,237]. While these alterations support clonal evolution and therapy resistance, they also create exploitable vulnerabilities. Targeting key DDR regulators, including ATM, ATR, CHK1, CHK2, and PARP1, presents a promising strategy to selectively weaken GSCs and enhance the efficacy of RT [238,239,240]. Another mechanism in GSCs’ radioresistance is the elevated expression of the DDR protein RAD51, which enables efficient repair of RT-induced double-strand breaks (DSBs). Targeting RAD51 with inhibitors like RI-1 and B02 impairs DDR, which enhances radiosensitivity and depletes the GSC population [125].

## 5. Perspectives and Future Directions

GSCs pose a significant challenge due to their intrinsic heterogeneity, plasticity, and dissemination in perinecrotic and invasive niches. The plastic behavior of these cells allows them to enter a quiescent and dormant state, making them resistant to conventional therapies.

Stem cell-based therapies are emerging approaches that hold promise. They may overcome the above-mentioned limitations thanks to their capability to penetrate deep hypoxic zones and eradicate GSCs regardless of their proliferative status or phenotypic subtype. The most commonly explored therapeutic cell types include engineered NSCs [241], mesenchymal stem cells (MSCs) [242], and hematopoietic stem and progenitor cells (HSPCs) (Table 2). What makes these cells attractive is their intrinsic tissue-specific trafficking ability, which enables them to home to sites of injury and target confined cell populations [243]. In the context of GBM, MSCs, and NSCs can migrate across the blood–brain barrier and selectively localize within the tumor microenvironment, including hypoxic and deeply infiltrative glioma regions [241,244]. This tumor-tropic behavior is largely governed by growth factors, chemokine signaling, and interactions with inflammatory cues in the microenvironment, leading to stem cell accumulation in tumors [244,245,246,247]. These cells are also attracted to hypoxic areas, which offers a solution to targeting quiescent GSCs residing in regions of the tumor that are otherwise difficult to access [248,249]. Additionally, by mimicking pericyte behavior, MSCs display perivascular migration, allowing them to integrate into the tumor’s vascular network and effectively navigate through the tumor mass [250]. This active tumor-homing ability enhances stem cells’ localization and persistence within the glioma microenvironment. Furthermore, cell-based therapies do not only rely on targeting specific tumor-associated antigens but instead utilize their innate homing properties, which provide an additional advantage in overcoming the cellular and molecular heterogeneity of GBM subpopulations [251].

Engineered stem cells provide a robust delivery platform for gene therapy due to their immune-evasive properties and relative ease of genetic modification. This is especially critical in GBM, where the clinical potential of gene therapy is undermined by significant delivery barriers, including the rapid degradation of free nucleic acids and their inefficient penetration through cellular membranes. Suicide gene therapy is a cancer treatment strategy in which mesenchymal stem cells (MSCs) are genetically modified to express “killer” proteins like Herpes Simplex Virus Thymidine Kinase (HSV-TK). HSV-TK encodes an enzyme that activates prodrugs, such as ganciclovir, converting them into cytotoxic metabolites that cause localized cell death. The importance of the suicide mechanism is to trigger self-destruct upon prodrug administration, minimizing immunological risks and potential oncogenesis associated with the use of exogenous stem cells. The effect is not restricted to MSCs but extends to neighboring tumor cells through the diffusion of toxic metabolites or via gap junctions (bystander effect) [252,253]. NSCs have been engineered to express dual-suicide genes, such as HSV-TK and cytosine deaminase (CD), which activate ganciclovir and 5-fluorocytosine (converted into 5-fluorouracile), respectively. This strategy offers synergistic cytotoxic effects, reduces the risk of treatment resistance or escape, and adds superior safety by efficiently self-eliminating the therapeutic cells after treatment [254]. Another dual-suicide gene therapy approach using bone marrow-derived mesenchymal stem cells (BMSCs) has been developed to target glioblastoma. BMSCs were designed to produce an anti-angiogenic protein (K5) and a radioisotope-absorbing protein (NIS), which is activated by radiation. The therapy reduced tumor size, inhibited blood vessel formation, and selectively eliminated the therapeutic BMSCs, as confirmed by real-time imaging [255]. Hematopoietic stem cells (HSCs) have also been used and engineered to deliver a TGFβ-blocking protein specifically within the GBM microenvironment using a tumor-specific MMP14 promoter. Combined with radiation therapy, this approach significantly reduced tumor growth, prolonged survival, and triggered a durable immune response in mice, highlighting its clinical promise [256]. Another therapeutic approach involves using MSCs to deliver oncolytic viruses (OVs) that reactivate key tumor suppressors like p53 and PTEN. This strategy protects OVs, facilitating viral replication and improving intratumoral dissemination [257]. Engineered NSCs were also used to deliver conditionally replicating oncolytic adenoviruses directly to glioblastoma tumors and have been clinically tested in both newly diagnosed and recurrent high-grade glioma patients [258].

A growing body of evidence emphasizes the CXCL12–CXCR4 axis as a central regulator of tumor cell migration, dormancy, and therapeutic resistance in GBM, making it a promising target for next-generation therapies. NSCs have been shown to migrate across the brain parenchyma through chemotactic signals, particularly in response to SDF-1α (CXCL12) secreted by astrocytes and endothelial cells at injury sites. This migratory behavior is mediated by CXCR4 expression in NSCs [243]. Elevated levels of SDF-1α are secreted in GBM necrotic and angiogenic niches, and the expression of its receptor CXCR4 has been found to correlate with glioma histological grade and invasive potential [259]. Using this feature, the tumor-homing capacity of human umbilical cord blood-derived MSCs was enhanced by overexpression of CXCR4. These modified MSCs exhibited superior tropism toward glioma tissues, particularly within hypoxic and chemokine-rich niches [246]. Notably, under temozolomide-induced stress, the CXCL12–CXCR4 axis modulates GSCs plasticity by altering transcriptional programs involved in quiescence and reactivation, thus playing a critical role in treatment resistance and tumor relapse [260]. Disrupting this dormancy-supportive niche represents another promising strategy. Clinical efforts using plerixafor, a CXCR4 antagonist, have demonstrated efficacy in mobilizing dormant leukemia and myeloma stem cells and sensitizing them to chemotherapy [261]. These findings suggest the dual therapeutic value of the CXCL12–CXCR4 axis, both as a molecular target to disrupt protective niches and as a navigational cue to guide engineered stem cells as delivery vehicles for precision therapy against dormant and treatment-resistant GSC populations.

Human induced pluripotent stem cells (hiPSCs) offer an alternative to traditional stem cell therapies like MSCs and NSCs, which, despite their tumor-homing abilities, are limited by limited proliferation and differentiation potential and challenges in genetic modification. hiPSCs, by contrast, provide unlimited self-renewal, patient-specific compatibility, and greater potential for precise genetic engineering. Their capacity to differentiate into neural lineages makes them suited for glioblastoma therapy. Notably, genome-edited neural stem and progenitor cells derived from hiPSCs have demonstrated superior efficacy in targeting and suppressing GSCs through the HSV-TK suicide gene approach, outperforming MSC-based approaches [262,263].

## 6. Conclusions

Recent technological advances have sharpened our understanding of GSCs’ complexity. The remaining challenges lie in translating this knowledge into effective therapies to outpace the aggressive evolution of glioblastoma. GSCs are the primary drivers of recurrence due to their capacity to proliferate and operate state changes to resist treatment, making them a critical target for therapeutic intervention. Conventional approaches often fail to eliminate GSCs or penetrate the blood–brain barrier (BBB) effectively. In contrast, engineered stem cell therapies, especially those utilizing human iPSCs, offer a compelling alternative. These platforms combine tumor-homing capabilities with precise genetic modifications, allowing for targeted and regulated delivery of therapeutic agents directly to GSCs, even within hypoxic zones. As such, integrating hiPSC-based technologies with our growing molecular understanding of GSC plasticity could represent a transformative step toward personalized and effective glioblastoma treatment.

## Figures and Tables

**Table 1:** Experimental drugs targeting GSCs.

Drug Name	Molecular Targets	Reference
Sorafenib	VEGFR, PDGFR, Raf kinase, c-KIT, and FLT3	[63,64,65,66,67]
Cabozantinib	MET, RET, AXL, VEGFR2, FLT3, and c-KIT.	[52]
Dasatinib	BCR-ABL, Src, c-KIT, and PDGFR	[53]
Nintedanib	PDGFRα + β), VEGFR1-3 and FGFR1-4	[54,55]
Pazopanib	VEGFR, PDGFR and c-KIT.	[56]
Pexidartinib	c-KIT, FLT3, and CSF-1/PDGFR	[57,58]
Ponatinib	VEGFR, PDGFR, FGFR, c-KIT, RET, and Src	[59]
Regorafenib	VEGFR1-3, PDGFR, FGFR, c-KIT, RET, and RAF	[60]
Sunitinib	VEGFR, PDGFR, and FLT3	[61]
Vandetanib	RET, VEGFR, and EGFR.	[62]
Depatuxizumab mafodotin	EGFR	[68]
Erdafitinib	FGFR	[69]
Erlotinib	EGFR	[70,71,72,73,74]
Dacomitinib	EGFR	[75,76,77]
Lapatinib	EGFR	[78]
Osimertinib	EGFR	[79,80,81]
Pemigatinib	FGFR1-3	[82]
AZD4547	FGFR1-3	[83]
NVP-BGJ398 (Infigratinib)	FGFR1-4	[84]
Futibatinib (TAS-120)	FGFR1-4	[85]
E-7090	FGFR1-3	[86]
Roblitinib (FGF-401)	FGF4	[87]
H3B-6527	FGFR4	[88]
Fisogatinib (BLU-554)	FGFR4	[89]
LY2874455	Pan FGFR	[90]
FIIN-1 (FGFR irreversible inhibitor-1)	FGFR1	[91]
PD173074	FGFR1	[92]
V4-015	FGFR4	[93]
BLU-9931	FGFR4	[94]
Capmatinib	HGFR	[95], NCT02386826
Crizotinib	ALK and c-MET	[96,97]
Dactolisib	PI3K/mTOR	[98,99], NCT02430363
Voxtalisib	PI3K/mTOR	[100]
Paxalisib	PI3K/mTOR	[101,102,103,104]
Buparlisib (BKM120)	PI3K	[105,106]
Pilaralisib	PI3K	[100]
Pictisilib	PI3K	[107]
Sonolisib (PX-866)	PI3K	[108]
NCT01458067	PIK3K (p110β isoform-specific)	NCT01458067
Everolimus	mTORC	[109]
Temsirolimus	mTORC	[110,111]
Decitabine	DNMT	[112,113]
5-azacytidine	DNMT	[114]
Balinostat	HDAC	[115]
Panobinostat	HDAC	[116]
Vorinostat	HDAC	[117]
Valproic acid	HDACI/IIa	[118,119,120]
KA2507	HDAC6	NCT03008018 [121]
JBI-802	HDAC6	NCT05268666
Melatonin	ABCG2/BCRP	[122]
Perifosine	BRCA1	[123]
GSI-1	NOTCH	[124]
Cyclopamine	SMO/SHH	[124]
RI-1	RAD51	[125]
B02	RAD51	[125]

**Table 2:** Clinical trials involving engineered stem cells.

Cell Type	Targeting Cells	Drug	Mechanism	Targeted Tumors	Clinical Phase (Status)	Study ID
NSCs	HB1.F3.CD NSCs	5-FC (Prodrug)	Inhibits TS (Suicide gene therapy)	Recurrent GBM	I (Completed)	NCT02015819
	HB1.F3.CD NSCs	5-FC (Prodrug)	Inhibits TS (Suicide gene therapy)	Recurrent GBM	I (Completed)	NCT01172964
	NSC-CRAd-S-pk7	CRAd-S-pk7 virus	Oncolytic adenovirus (Virotherapy)	Newly diagnosed GBM	I (Completed)	NCT03072134
	NSC-CRAd-S-pk7	CRAd-S-pk7 virus	Oncolytic adenovirus (Virotherapy)	Recurrent GBM	I (Recruiting)	NCT05139056
	hCE1m6-NSCs	Irinotecan (Prodrug)	Inhibits Topo IA (Suicide Gene therapy)	Recurrent GBM	I (Active, not recruiting)	NCT02192359
MSCs	BM-MSCs	Ad5-DNX-2401	Oncolytic adenovirus (Virotherapy)	Recurrent GBM	I (Recruiting)	NCT03896568
	A-MSCs	/	Dose-escalation study of A-MSCs	Recurrent GBM	I (Recruiting)	NCT05789394
	MSC-CD	5-FC (Prodrug)	Inhibits Thymidine Synthase (Suicide gene therapy)	Recurrent GBM	I/II (Completed)	NCT04657315
HSPCs	HPCs—P140K MGMT Lentiviral Vector	O6-benzylguanine	MGMT inhibition (Tumor chemosensitization)	GBM	II (Recruiting)	NCT05052957
	PBSCs—P140K MGMT Lentiviral Vector	O6-benzylguanine	MGMT inhibition (Tumor chemosensitization)	GBM	I (Completed)	NCT01269424
	HSPCs—Interferon-α2	/	Enhance immune response (Immunomodulation)	GBM	I/II (Recruiting)	NCT03866109

5-FC: 5-fluorocytosine; Topo IA: topoisomerase I; NSCs: neural stem cells, PBSCs: peripheral blood stem cells; BM-MSCs: bone marrow-derived mesenchymal stem cells; A-MSCs: adipose-derived mesenchymal stem cells; CD: cytosine deaminase; HSPCs: hematopoietic stem and progenitor cells.

## Data Availability

No new data were created or analyzed in this study.
